# Detecting frail, older adults and identifying their strengths: results of a mixed-methods study

**DOI:** 10.1186/s12889-018-5088-3

**Published:** 2018-01-30

**Authors:** Sarah Dury, Eva Dierckx, Anne van der Vorst, Michaël Van der Elst, Bram Fret, Daan Duppen, Lieve Hoeyberghs, Ellen De Roeck, Deborah Lambotte, An-Sofie Smetcoren, Jos Schols, Gertrudis Kempen, G.A. Rixt Zijlstra, Jan De Lepeleire, Birgitte Schoenmakers, Dominique Verté, Nico De Witte, Tinie Kardol, Peter Paul De Deyn, Sebastiaan Engelborghs, Liesbeth De Donder

**Affiliations:** 1 Department of Educational Sciences, Vrije Universiteit Brussel, Pleinlaan 2, 1050 Brussels, Belgium; 2 Department of Clinical and Lifespan Psychology, Vrije Universiteit Brussel, Pleinlaan 2, 1050 Brussels, Belgium; 3 Department of Health Services Research, Care and Public Health Research Institute (CAPHRI), Maastricht University, Duboisdomein 30, 6229 GT Maastricht, The Netherlands; 4 Department of Public Health and Primary Care University of Leuven, Kapucijnenvoer 33 blok J postbus 7001, 3000 Leuven, Belgium; 5 Faculty of Education, Health and Social Work, University College Ghent, Keramiekstraat 80, 9000 Ghent, Belgium; 6 Laboratory of Neurochemistry and Behavior, University of Antwerp, Universiteitsplein 1, DT.652, 2610 Antwerp, Belgium; 7 Department of Health Services Research, Care and Public Health Research Institute (CAPHRI), Maastricht University, P.O. Box 616, 6200 MD Maastricht, The Netherlands; 8 Department of Family Medicine, Care and Public Health Research Institute (CAPHRI), Maastricht University, P.O. Box 616, 6200 MD Maastricht, The Netherlands; 9 Laboratory of Neurochemistry and Behaviour, University of Antwerp, Universiteitsplein 1, 2610 Wilrijk, Belgium; 10 Research Foundation Flanders (FWO), Egmontstraat 5, 1000 Brussels, Belgium

**Keywords:** Frail elderly, Caregivers, Social participation, Health literacy, Independent living, Quality of life, Belgium, Surveys and questionnaires, Residence characteristics

## Abstract

**Background:**

The debate on frailty in later life focuses primarily on deficits and their associations with adverse (health) outcomes. In addition to deficits, it may also be important to consider the abilities and resources of older adults. This study was designed to gain insights into the lived experiences of frailty among older adults to determine which strengths can balance the deficits that affect frailty.

**Methods:**

Data from 121 potentially frail community-dwelling older adults in Flemish-speaking Region of Belgium and Brussels were collected using a mixed-methods approach. Quantitative data were collected using the Comprehensive Frailty Assessment Instrument (CFAI), Montreal Cognitive Assessment (MoCA), and numeric rating scales (NRS) for quality of life (QoL), care and support, meaning in life, and mastery. Bivariate analyses, paired samples t-tests and means were performed. Qualitative data on experiences of frailty, frailty balance, QoL, care and support, meaning in life, and mastery were collected using semi-structured interviews. Interviews were subjected to thematic content analysis.

**Results:**

The “no to mild frailty” group had higher QoL, care and support, meaning in life, and mastery scores than the “severe frailty” group. Nevertheless, qualitative results indicate that, despite being classified as frail, many older adults experienced high levels of QoL, care and support, meaning in life, and mastery. Respondents mentioned multiple balancing factors for frailty, comprising individual-level circumstances (e.g., personality traits, coping strategies, resilience), environmental influences (e.g., caregivers, neighborhood, social participation), and macro-level features (e.g., health literacy, adequate financial compensation). Respondents also highlighted that life changes affected their frailty balance, including changes in health, finances, personal relationships, and living situation.

**Conclusion:**

The findings indicate that frailty among older individuals can be considered as a dynamic state and, regardless of frailty, balancing factors are important in maintaining a good QoL. The study investigated not only the deficits, but also the abilities, and resources of frail, older adults. Public policymakers and healthcare organizations are encouraged to include these abilities, supplementary or even complementary to the usual focus on deficits.

## Background

Many western countries, including Belgium, have aging populations with multiple chronic health problems, such as diabetes, respiratory and cardiovascular diseases [1]. Policymakers are attempting to develop strategies to meet these challenges [2], such as motivating (frail) older people to live at home for as long as possible [3, 4] and to follow “healthy and active aging” principles [5]. Living at home for as long as possible is also often preferred by (frail) older people [6]; however, effective measures to identify frail, older adults in need of care and support are lacking [7]. Early detection and appropriate interventions for frail and vulnerable older people are essential to prevent unnecessary adverse outcomes [8–10], such as institutionalization [11], mortality [4], and falls [12].

Current research on frailty, in both the medical and social science literature, covers a wide range of definitions and descriptions [13]. Previously, most attention has been paid to biomedical aspects; this approach defines frailty as a physical construct or phenotype [14], often representing an accumulation of health deficits [15, 16]. However, a growing number of studies have explored frailty in terms of the experience of not only physical issues, but also psychological, social, cognitive, and environmental problems, stressing the need for a more multidimensional view of frailty [17–19]. In addition, older people, who are classified by others as frail, frequently do not identify themselves as such [20]. Thus, frailty requires a broader perspective in terms of measurement, detection, and intervention strategies.

The first “modern” textbook of geriatrics was published in 1973, edited by John Brocklehurst. Against this background, Rockwood and colleagues [16] built on the ideas of Brocklehurst [21] to conceptualize a “dynamic model of frailty”, a state that arises from a dynamic interplay between a variety of factors. This model emphasizes the presence of multiple interacting factors, as well as complex relationships within and between deficits and also resources and abilities [16, 22]. Using the same line of reasoning, Sipsma [23] called for attention to be paid to the development of a “gerodynamic model”, described as an approach to understand the balance between losses and deficits on one side, and support and autonomy on the other side. Two individuals with the same level of frailty, for instance, can have a different “frailty balance” because of the kinds of support they have [24].

Regarding a more positive frailty balance, several studies have shown that frail, older adults can have satisfying lives, despite their deficits [25]. For example, physical and social frail older adults experiencing physical and/or social negative changes maintained equal levels of psychological well-being over time [26]. Similarly, a Dutch study showed that almost half of multidimensional (physical, social, cognitive and psychological) frail participants reported a good to excellent quality of life (QoL) [27]. Despite this, research on frailty mainly focuses on associations between frailty and adverse (health) outcomes (e.g. [28–31]), such as increased risk of premature mortality, hospitalization, institutionalization, falls, and comorbidities [32, 33] and decreased well-being [34]. Consequently, positive outcomes may be overlooked. In light of active aging, it is particularly important to identify the resources and the intrinsic power (i.e., intrinsic abilities, skills, and competences) older people possess as well, instead of focusing only on deficits [35].

Such an approach could enable a paradigm shift in prevention and intervention strategies, away from decreasing frailty and towards reinforcing strengths and restoring the frailty balance; however, insight into which resources and intrinsic power frail older adults have that might balance their frailty is lacking. Hence, there is a need to develop an inventory of what we term “balancing factors”, i.e., resources for meeting particular psychological, social, physical, environmental, and/or cognitive challenges.

Since January 2015, 21 researchers from the University of Antwerp, Vrije Universiteit Brussel, University College Ghent, the Catholic University of Leuven (Belgium), and Maastricht University (the Netherlands) have been working on the D-SCOPE project, which stands for Detection, Support and Care for Older people – Prevention and Empowerment. The project, which continues until December 2018, aims (1) to identify strategies for proactive detection community-dwelling older people at risk of frailty; and (2), to guide them towards appropriate support and/or care, with a focus on empowerment. The goals of the present paper were threefold, and focused on a strengths-based approach to aging. First, we aimed to examine how frail, older adults perceive their frailty, QoL, care and support, meaning in life, and mastery (as in mastering their own situation and being in control of the situation they live in). Second, we aimed to identify balancing factors that might influence the relation between frailty and positive outcome variables. The third objective was to explore which life changes and turning points older people experience and how these affect their frailty, QoL, care and support, meaning in life, and mastery.

## Methods

### Multi-actor, mixed-methods study design

The D-SCOPE comprises three research phases: 1) development of multidimensional frailty risk profiles; 2) identification of balancing factors and positive outcomes; and 3) development of a frailty balance assessment instrument and intervention. The first phase, in which frailty risk profiles of community-dwelling older persons were identified, was completed using data from a cross-sectional sample (*N* = 28,049) from the Belgian Ageing Studies project (see [36]). The second phase is detailed in this paper, and involved data on 121 community-dwelling older people at risk of frailty, collected between November 2015 and March 2016 using a multi-actor and mixed-methods approach. The third and final phase of the D-SCOPE project started in March 2017 and will end in April 2018.

### Ethical approval and informed consent

This study was approved by the Human Sciences Ethical Commission of the Vrije Universiteit Brussel (file number ECHW_031). For this study, the participants were asked to sign an informed consent agreement. Respondents were informed about the voluntary nature of their involvement in the study, their right to refuse to answer any questions, and the privacy of their responses. Respondents had the right not to participate in the study and to withdraw their consent at any time. They were informed that they could discontinue their participation during the study without giving a reason and without negative consequences. Refusal to consent led to exclusion from the study. All data were anonymous and analyzed according to the rules of the Belgian Privacy Commission [37].

### Target population and sample recruitment

A purposive sampling procedure was used to identify, recruit, and select potentially frail community-dwelling adults aged 60 years and over in the Flemish-speaking part of Belgium and Brussels. Five homecare organizations recruited 64 respondents from among their clients, and a further 57 respondents were recruited by snowball sampling.

All participants were screened based on the inclusion and exclusion criteria before and during the face-to-face interview. With regard to inclusion, the selection of participants was based on risk profiles for multidimensional frailty, which included age, gender, marital status, level of education, household income, whether the participants had moved in the previous 10 years, and their country of birth [36]. The exclusion criteria were hospitalization and any state that may interfere with a good understanding of the questions (referring to being too sick to participate in the interview, too exhausting to participate, …) (according to the participant or an informal caregiver), or the inability to provide adequate answers during the face-to-face interview (as noted by the interviewer). Also, the presence of dementia served as an exclusion criterion. Therefore, the homecare organizations involved in the recruitment process were instructed to exclude people that received a dementia diagnosis, as determined by a doctor (specialist or general practitioner). Respondents recruited through snowball sampling whom mentioned to be diagnosed with dementia were removed out of the analysis as well. Examples of people whom were unable to provide adequate answers are as follows: people who did not understand the questions, or ignored/did not respond to the questions, but rather told things they were preoccupied with (i.e. illness of other family members).

Eligible participants received written information on the study and an informed consent form for participation in the study in their preferred language (Dutch or French). If general language problems or difficulties with the specific preferred language made reading the written information and consent form impossible, a caregiver, social worker, or researcher provided the information to the potential participant. To achieve maximum participation of participants with a background of migration, an interpreter attended the interviews when necessary (*n* = 12). The interview schedule was also available in Dutch and French. If participants were not capable of signing this document, a family member or legal representative was allowed to sign it on their behalf, as stipulated in the Belgian civil code. No incentives were offered for study participation. Interviews were conducted in the homes of the participants.

## Measures

### Quantitative data collection

#### Socio-demographic and economic characteristics

Specific individual characteristics established as related to multidimensional frailty [36] were assessed: age, gender, marital status, level of education, household income, whether the participants had moved in the previous 10 years, and country of birth.

##### Multidimensional frailty

Frailty was measured using the Comprehensive Frailty Assessment Instrument (CFAI) [17], b), which is a self-administered instrument, that measures four domains of frailty. For the physical domain, the respondent’s general physical health was assessed (four items, e.g., walking up a hill or stairs). The psychological domain was captured by measuring mood disorders and emotional loneliness (eight items, e.g., losing self-confidence). The social domain was evaluated based on social loneliness (three items, e.g., “There are enough people who I feel close to”) and potential social support network (ten items, e.g., partner, children, and neighbors). Finally, environmental frailty was assessed based on factors related to the suitability of the physical housing environment (five items, e.g., insufficient comfort in the house). Scores for each domain, which theoretically range from 0 to 100, were calculated by adding the scores for the specific items. The total CFAI score was calculated by summing the scores for each domain. After the study was completed, a fifth domain was added to the total CFAI-plus score [38]: subjective cognitive frailty (see below for more information). Higher scores indicated more frailty. The total CFAI score was used to identify three levels of frailty: 1) no to mild frailty, 2) moderate frailty, and 3) severe frailty.

Cognitive frailty was not included in the original CFAI; however, for the purpose of this study, we added four supplementary questions to the CFAI, to assess cognitive frailty [38], and the answers to these questions were used in the calculation of the total adapted CFAI score, namely CFAI-plus. These four questions were selected from the Dutch version of the Informant Questionnaire on Cognitive Decline in the Elderly (IQCODE-N) [39], and were found to be valid and reliable [38].

##### Cognitive impairment

In addition, objective cognitive frailty was measured using the Montreal Cognitive Assessment (MoCA), which is a brief cognitive screening tool designed to detect mild cognitive impairment or mild dementia [40]. The MoCA consists of 12 items and examines multiple domains of cognitive function, including short-term memory, visuospatial ability, executive function, attention, language, and orientation in time and place. In comparison with the Mini Mental State Examination (MMSE) [41], the MoCA assesses a broader range of cognitive domains and displays greater sensitivity for detection of mild cognitive impairment [42]. To correct for education effects, one point was added for participants with 12 or fewer years of education [40]. Total scores ranged from 0 to 30; higher scores indicate better objective cognition.

##### Positive outcome measures

Finally, QoL, care and support, meaning in life, and mastery were assessed with 1-item questions developed as part of the D-SCOPE project, e.g., “On a scale from 0 to 10, to what extent do you feel that your life is meaningful (worthwhile, useful, having desires), that you are looking or striving for something?” The scores ranged from 0 (bad) to 10 (excellent) on a numerical rating scale [43]. These 1-item questions were used to gain insight into the ‘overall’ experience regarding QoL, sufficiency of care and support, meaning in life, and mastery. Whereas QoL has been measured on a scale from 0 to 10 in previous studies (i.e. [44–46]), we choose to measure the other constructs in this way as well due to the vulnerable population we were dealing with, and enabling comparability between the measures.

To assess the significance to the participant of each score, each answer was followed by a question to discern whether the participant perceived the score as poor, average, or good. Additionally, for QoL, care and support, meaning in life, and mastery, each participant provided a score for their present situation, the situation 1 year earlier (retrospective), and the expected situation 1 year later (prospective). Each answer was also followed by a question to discern whether the participant perceived the score as poor, average, or good.

### Qualitative data collection

Following quantitative data collection, the same researchers conducted semi-structured interviews (with open-ended questions) with the participants. The topic list consisted of four main questions: (a) “How do you experience frailty and what does frailty mean to you?”; (b) “How does you experience of frailty have an effect on your QoL, care and support, and meaning in life, and to what extent does frailty control the things happening in your life?”; (c) “What should an older person do, have, or need to maintain his/her quality of life when becoming frail?”; (d) “What were the highlights and what were the low points in life during the past year, did changes occur? And how do you feel about the future?” The topic list was developed by the D-SCOPE research group, which consists of researchers specialized in gerontology and/or frailty and represents several disciplines (e.g., geriatric medicine, psychology, educational sciences, etc.). A panel of experts approved all the questions, helping to ensure the content validity of the interviews [47]. The expert panel consisted of two neurologists specializing in dementia, a psychologist specializing in neuropsychology and dementia, five adult education scientists specializing in social gerontology, three general practitioners specializing in frailty in later life, and two social gerontologists specializing in public health. Moreover, the researchers who conducted the interviews received training consisting of four steps: 1) explanation and discussion of the study protocol; 2) explanation and exercises on administering the MoCA (led by a psychologist); 3) debriefing regarding the instructions for the translated questionnaires; 4) practice conducting the interviews with simulated patients while being recorded. Each researcher practiced the interview with three simulated patients.

All the interviews were held in the language of the respondent’s choice, and, with each respondent’s permission, they were digitally recorded with Audacity (Dominic Mazzoni and Roger Dannenberg, Pittsburgh, Pennsylvania, USA), and subsequently transcribed verbatim. During the data collection period, the head of the research team gave general guidance and specific advice in cases involving participant questions and/or difficulties, and was onsite during each researcher’s first interview to observe and redirect when necessary.

After conducting the simulated interviews, the recorded interviews were analyzed by a group consisting of the head of the research team, two professors with expertise in qualitative interviewing and vocational training, the researchers, and the simulated patients. Based on these analyses, the interview guidelines were completed and scenarios were added to address newly identified potential difficulties. All researchers received a list of the definitions of all the terms used in the questionnaire.

The variables collected throughout the study, both quantitative and qualitative, are presented in Table 1.

**Table 1:** Study variables quantitative survey and qualitative interview

Domain	Variables/scale	Description	Older people	Informal caregivers	GP
Quantitative survey					
Socio-demographics	Date of birth (age)		x	x	x
	Gender		x	x	x
	Nationality		x	x	
	Country of birth		x	x	
	Marital status		x	x	
	Living arrangement		x	x	
	Practice	e.g. solo or group practice			x
Frailty	Comprehensive Frailty Assessment Instrument (CFAI) (De Witte et al., 2013)	27 items, 4 domains (physical, psychological, social, environmental)	x		
	IQCODE-N (Jonghe & Schmand, 1996)	Subjective cognitive frailty	x		
	‘Clinical judgment’	1 item, 10-point scale for each domain		x	x
Objective cognitive frailty	Montreal Cognitive Assessment (Nasreddine et al., 2005)	12 items, 30-points, multiple domains: memory (learning and delayed recall), visuospatial abilities, executive functioning, attention, concentration, working memory are language, orientation to time and place	x		
Competences older people	Quality of life	1 item, 10-point scale	x	x	x
		2 items, 10-point scale, rating for past (one year ago), and future (one year ahead)	x		
		1 item, qualification of the number (poor, average or good)			
	Care and support	1 item, 10-point scale	x	x	x
		2 items, 10-point scale, rating for past, and future	x		
		1 item, qualification of the number (poor, average or good)			
	Meaning in life	1 item, 10-point scale	x	x	x
		2 items, 10-point scale, rating for past, and future	x		
		1 item, qualification of the number (poor, average or good)			
	Mastery	1 item, 10-point scale	x	x	x
		2 items, 10-point scale, rating for past, and future	x		
		1 item, qualification of the number (poor, average or good)			
Main topics/themes discussed in qualitative interviews					
Frailty		e.g. *‘As people age, it is said that they become frail? How do you experience this yourself?’*; *‘What does frailty mean to you?’*			
Outcomes	Quality of life	e.g. ‘*Do you think/believe that frailty affects your quality of life?*’; ‘*What are the things that contribute to your quality of life, despite being frail?*’			
	Care and support	e.g. *‘What is the role of caregivers to maintain a qualitative life?’*			
	Meaning in life	e.g. ‘*What makes your life meaningful?’*			
	Mastery	e.g. *‘Do you feel like you have control over the things that happen in your life?’*			
Balancing factors		e.g. ‘*What should an older person do to maintain his/her quality of life / mastery / meaning in life when becoming frail?*’			
Life-events and turning points	History	e.g. ‘*Can you describe important positive and negative changes that happened in the past year?*’			
	Future perspective	e.g. ‘*Do you think your life might change within a year? Are there dreams you want to accomplish?’*			

### Data analyses

Quantitative and qualitative data were analyzed separately [48]. Regarding the quantitative data, bivariate analyses (paired samples t-tests with Bonferroni correction) were performed to assess the relationships between the independent variables (i.e. sociodemographic and socioeconomic characteristics) and mean scores and standard deviations were calculated for each frailty domain, multidimensional frailty, and other outcome variables included in our study. All statistical analyses were carried out using SPSS software version 24 (SPSS Inc., Chicago, IL, USA).

Qualitative interviews were subjected to thematic content analysis using an adapted version of the Qualitative Analysis Guide of Leuven (QUAGOL) [49]. This guide comprises two main processes: a preparatory coding process and an actual coding process. During the preparatory coding process, each researcher first read a series of interviews. After the experiences and findings were discussed, an initial conceptual interview scheme was developed. This stage was followed by a fitting-test of the conceptual interview scheme, in which a single interview was coded and discussed by all researchers. During the actual coding process, all the interviews were divided among the researchers (each interview was subject to analyses by two researchers) and coded using MAXQDA (VERBI Software, Berlin, Germany) software. The codes of each researcher were evaluated and compared, and when necessary, the findings were discussed until consensus was reached. All data were anonymized.

## Results

### Sample characteristics

The descriptive statistics of the participants are presented in Table 2. The mean age of study participants was 78.8 years (range 60–95 years). The majority were women (62.8%) and widowed (50.4%). 14% of the participants had a migration background and 90.9% had the Belgian nationality. The mean score on the MoCA was 21 (range 0–30).

**Table 2:** Sociodemographic and socioeconomic characteristics of the participants (*N* = 121)

	M	SD	%	N*
Age (years)	78.8	8.6		120
Female			62.8	76
Migration background			14.0	17
European			7.4	9
Non-European			6.6	8
Nationality				
Belgian			90.9	110
Other European			5.0	6
Non-European			4.1	5
Marital status				
Married			28.9	35
Never married			7.4	9
Divorced			12.4	15
Widowed			50.4	61
Cohabiting			0.8	1
Cognition				
MoCA	21	4.7		110
MoCA with correction	22	4.5		104

Participants of Turkish nationality/migration background were categorized as Non-European

*M* Mean, *SD* Standard deviation, *N* Number of participants. The number of participants changed according to the number of missing answers due to non-response to individual questions, *MoCA* Montreal Cognitive Assessment

### Quantitative analysis results

#### Frailty

Self-perceived frailty, described as the total CFAI score and the five frailty domain scores is detailed in Table 3. The participants in this study were a heterogenic group, which can be seen in for example the high standard deviations of the mean scores per frailty domain, which were highest for physical and psychological frailty. This means that participants score both way above and below the mean score, which might be explained by the fact that the different risk factors for frailty, based on which most of the participants were selected, differ per frailty domain [36], referring to multidimensional frailty. The mean physical frailty score was the highest (51.3); however, this score had a high standard deviation (37.5). The mean environmental frailty score was the lowest (18.1, SD = 17.1). Regarding the different frailty domains, it appeared that over half of the respondents had severe cognitive frailty (53.8%), while few had severe social frailty (14.2%). Only 3.3% (*n* = 4) of the participants were non-frail in all domains, and 11.6% (*n* = 14) were frail in every domain.

**Table 3:** Self-perceived frailty scores of the overall study group (*N* = 121) and frailty subgroups

				No to mild frailty		Moderate frailty		Severe frailty	
	*M*	SD	*N* (*m*)	%	*N*	%	*N*	%	*N*
Physical frailty	51.3	37.5	119 (2)	39.5	47	32.8	39	27.7	33
Psychological frailty	26.5	23.2	117 (4)	53.0	62	23.1	27	23.9	28
Social frailty	40.3	19.0	120 (1)	53.3	64	32.5	39	14.2	17
Environmental frailty	18.1	17.1	119 (2)	34.5	41	47.9	57	17.6	21
Cognitive frailty	34.9	21.5	117 (4)	22.2	26	23.9	28	53.8	63
Total frailty (5 domains)	37.0	12.0	111 (10)	34.2	38	30.6	34	35.1	39

*M* Mean, *SD* Standard Deviation, *N* Number of participants, *m* missing

#### QoL, care and support, meaning of life, and mastery

The numeric ratings scale scores for QoL, care and support, meaning in life, and mastery, including ratings for the present situation, 1 year before, and 1 year ahead, are detailed in Table 4. Paired samples t-tests with Bonferroni correction were performed. For all outcome variables, mean scores ranged between 7.8 and 8.1. Specifically, the participants scored high for QoL (M = 7.8, SD = 1.6), care and support (M = 8.0, SD = 2.2), meaning in life (M = 8.0, SD = 1.8), and mastery (M = 8.1, SD = 1.8). A small subgroup had scores of 5 or lower for each outcome variable: 7.9% for QoL, 8.9% for care and support, 8.7% for meaning in life, and 9.6% for mastery. For all four outcome variables, scores for 1 year earlier were similar to the current scores, but the scores for the next year were slightly lower compared to the current scores. On average, participants experienced significantly greater quality of life in the present (*M* = 7.69, *SD* = 1.52) than in the future (*M* = 7.41, *SD* = 1.83). There were no significant differences between quality of life in the present and past. For care and support, participants experienced significantly greater care and support in the present (*M* = 8.11, *SD* = 1.84) than in the future (*M* = 7.80, *SD* = 2.07). There were no significant differences between care and support in the present and past. For meaning in life the same picture occurs. Significantly greater meaning in life in the present (*M* = 7.93, *SD* = 1.79) than in the future (*M* = 7.74, *SD* = 2.09). There were no significant differences between meaning in life in the present and past. Finally, for mastery, significantly greater sense of mastery is experienced in the present (*M* = 8.10, *SD* = 1.77) than in the future (*M* = 7.83, *SD* = 1.89). There were no significant differences between sense of mastery in the present and past.

**Table 4:** QoL, care and support, meaning of life, and mastery scores among the study participants (*N* = 121)

	Present			1 year earlier			1 year later		
	M	SD	N	M	SD	N	M	SD	N
QoL	7.8	1.6	114	7.7	1.8	114	7.4	1.8	106
Care and support	8.0	2.2	112	7.9	2.0	109	7.8	2.1	100
Meaning in life	8.0	1.8	115	8.0	1.6	115	7.7	2.1	101
Mastery	8.1	1.8	115	8.0	2.0	114	7.8	1.9	101

*QoL* Quality of life, *M* Mean, *SD* Standard deviation, *N* Number of participants

#### Qualitative analysis results

Based on the 121 interviews, 5791 codes were generated; a summary of the results is presented below.

### Perceived frailty, QoL, care and support, meaning in life, and mastery

Frailty was delineated by older people in a broad and multidimensional way; however, they often felt less cognitively and physically frail than their peers: “*When I look at the people around me, with 77 or 78 years, I’m a fortune’s favorite*” (77-year-old woman), or they described their physical frailty as normal aging or being less fit than in their earlier years. Psychological frailty was seen as being more emotional, not being able to stand gossip, having depressive symptoms, and fearing institutionalization, hospitalization, or falling. Social frailty was experienced with loss of formal networks and shortage of informal networks. Environmental frailty was described as encountering problems in their home, in public areas, and on public transport, and feelings of insecurity.

Besides the frailty domains covered by the CFAI, the participants also mentioned experiencing problems with information and communications technology (ICT), finances, and ageism. The different frailty domains were mostly perceived as related and cumulative. This experience was well described by a 70-year-old widower who experienced physical, social, and environmental frailty simultaneously:

*“The absence of someone at your side. That’s the problem. I sit here at night and if I go to bed it is okay. But what if I can’t go upstairs? What do I have to do then? Stay downstairs? What do I have to do? One starts to think about that situation.”*

As for the positive outcomes, respondents reported that, overall, a good QoL could be established based on multiple aspects, such as health, community participation, (in)formal care, and social contact. Good quality of care and support was often a focus. Many of the respondents experienced good care provided by general practitioners, homecare nurses, home-helpers, and informal caregivers.

The participants described mastery as autonomy, signifying that they remained able to choose according to their own preferences, and had sufficient knowledge and capacity to continue making these choices. The desire to also maintain this mastery in care situations was expressed by a 66-year-old divorced woman:

*“I am not the subject that suffers here. I am… I would like to be the employer of assistants, including my cleaning lady, my GP, my physiotherapist. They are my team and I am the bandmaster.”*

As for meaning in life, the participants mostly expressed this as relating to a sense of coherence of past life events and finding purpose in activities with relatives and likeminded persons.

### Balancing factors influencing frailty, diverse positive outcome variables, and the association between frailty and the outcomes

The participants experienced that they possessed several balancing factors that could decrease their negative experiences of frailty and increase their positive outcomes. The balancing factors appeared to occur on three different levels: individual, environmental, and macro. Balancing factors on the individual level were personality traits, coping strategies, and resilience. Optimism, for example, was a positive personality trait in dealing with frailty and other aging problems. Many respondents experienced difficulties in their lives that resulted in a severe impact on their daily QoL; however, most of them applied coping strategies when facing these difficulties, such as acceptance of the situation, staying positive, and actively looking for support when needed. An 87-year-old widow expressed her situation as follows:

*“Because of this accident, I lost everything from what I used to do. But I say there are always happy days in life, however few there are.”*

Environmental factors refer to different aspects in the neighborhood or network of older people. First, living in a neighborhood with adequate resources is an important balancing factor. These resources include supportive public transport, walkability, and sufficient services and amenities close by. Living in a dynamic neighborhood where people have good contact with their neighbors appears to be an important balancing factor. Neighbors also appear to be important for providing support; however, people moving in and out of a neighborhood could create a negative balance. Second, having good social contacts with healthcare professionals and informal caregivers, in addition to receiving care, is an important balancing factor. Moreover, having high-quality relationships with family, friends, and neighbors that allow concerns and problems to be shared appears to be important in the context of frailty balance. An 89-year-old widow perceived her neighborhood as a balancing factor for maintaining her QoL:

*“When the weather’s fair, I can walk to my fence and back 10 times in an afternoon, with my little roly-car [rollator, i.e., a walking frame equipped with wheels], and that makes me feel good. Someone's bound to walk by, so you can have a bit of chit chat.”*

On the macro level, having an adequate retirement income helped to balance frailty in a positive sense. Lower dignity experienced by older people, lower health literacy, and not being able to follow digital changes in society balanced frailty in a negative sense. An 81-year-old married man explained how digital changes created anxieties:

*“The evolution of everything goes so fast that you can no longer follow. If you are now doing payments with the computer, it happens regularly that the bank changes their data or creates another website. That is not always easy.”*

### Life changes and turning points experienced by older people and effect on frailty, QoL, care and support, meaning in life, and mastery

We discovered that, not only did adverse life events (e.g., [50]) affect the participants’ frailty and life outcomes, but also positive life events (e.g., the birth of a grandchild or an operation) also affected their frailty in a positive way. In addition, some turning points were very sudden (e.g., the death of a partner), while others were rather described as “transition phases,” which occurred over a longer time.

These life changes and turning points can be classified in five domains. The first domain includes changes in the participant’s financial situation, such as financial losses (e.g., money stolen by grandchildren) and retirement, but also positive changes such as receiving a gift or bonus. The second domain describes changes in health. The frail, older adults experienced physical problems, other illnesses, and general physical deterioration, but also positive changes as a result of physical interventions. This was illustrated by the following comment from a 69-year-old widower:

*“A high point was that operation. It worked, and I could walk again. Being able to walk without a stick, that was great.”*

A third domain includes changes in personal relationships, such as divorce from a partner, a divorce between a child and their partner, and conflicts in the family. This domain also included the death or illness of a spouse or, positively, the birth of grandchildren. The fourth domain comprises changes in living situations, including moving to another house, relatives moving away (leading to loneliness), and the installation of devices in order to “age in place” (e.g., a stair lift). The final domain consists of all other changes such as loss of the ability to drive a car or becoming an informal caregiver.

## Discussion

This report presents an overview of a mixed-methods study within the larger D-SCOPE project, in which the results of quantitative and qualitative analyses shed light on (perceived) levels of frailty and the relationships between frailty and positive outcomes. Our study shows that frail, older adults still have a good QoL, generally report that they receive sufficient care and support, and have relatively high levels of meaning in life and mastery. Moreover, balancing factors at the individual (e.g., adapting to difficulties), environmental (e.g., social contacts), and macro (e.g., digital changes) levels have roles in influencing self-perception of frailty in older people, and how they rate the positive outcome variables. In addition, older people’s (perceived) frailty and positive outcomes are influenced by turning points and life events. A key finding in this study is that, in addition to negative turning points and life events (e.g., the death of loved ones), some older people also experience positive turning points and life events (e.g., the birth of a great-grandchild), which might influence their (perceived) frailty and outcomes in a more positive way. This study is one of the first to focus on strengths and resources among frail community-dwelling older people, rather than merely on deficits and dependencies. This strengths-based approach is (amongst other approaches) important because many older people dislike when only their deficits are taken into account [7]. In addition, this empowerment approach may encourage the participation of older adults in care decisions and promote positive health outcomes, as well as highlighting the potential for older adults to be active participants in decisions and actions that affect their QoL [51, 52].

Regarding the quantitative results, the data concerning the first research aim revealed that the majority of frail, older adults generally reported having a good QoL. This is in line with the findings of Puts et al. [53], whose qualitative study showed that 8 of 11 frail participants reported having a “good” QoL. It also corroborates the findings of Ament et al. [27] and Zaslavsky et al. [54], who found that nearly 50% of participants who were (at least) frail on the physical domain reported having a “good” to “excellent” QoL. In addition, a Swedish study established that 63.5%–74.4% of older people receiving help stated that they had a “good” or “very good” QoL, respectively, compared to 85.3%–93.8% among those without help [55]. Regarding the other positive outcome measures, the participants in our study reported that they received enough care and support and experienced relatively high levels of meaning in life and mastery. Moreover, only a small group of participants reported lower scores for the positive outcome variables. This might be surprising, as frailty is generally associated with adverse outcomes [56, 57]; however, these positive findings could be explained by the fact that not every older adult who is defined as frail, based on objective measures, actually perceives them self as frail (e.g., [58]). In addition, Netuveli and Blane [59] concluded that aging itself does not negatively influence positive outcomes, such as QoL.

Other plausible explanations may be deduced from the findings related to the second research aim, which demonstrated that frail, older adults possess several resources or, as we termed them, balancing factors. The qualitative analyses showed that some frail, older adults possess individual characteristics (such as certain personality traits, coping strategies, and resilience) that help them to deal with frailty and changing life events, which is consistent with previous research [60, 61]. At the individual level, these balancing factors refer to individual characteristics that people possess (e.g., actively looking for support), which fits perfectly within the concept of “Selection, Optimization, and Compensation” put forward by Baltes and Baltes [62]. This theory proposes that older people adopt a variety of strategies to enhance adaptation to their changing circumstances or limitations that arise in everyday life, and suggests that adaptation ensures that an activity (or parts of an activity) that is too demanding for an older individual may be reduced or stopped, and parts of an activity may be selected for optimization, so that they can still be performed.

There were also balancing factors apparent at the environmental level. For example, some participants mentioned that having social contacts with others, or being able to walk again with a rollator (which enabled them to go out and have social contacts), were important factors that allowed them to have a good QoL, despite being frail. From an environmental gerontology perspective, it has been suggested that older adults have a dynamic relationship with their place of residence and community [63]. In line with the results, previous research has indicated that the neighborhood provides socially supportive networks and appears to be a vital element in the support systems of older adults [64]; however, the accessibility of the home environment is an important precondition in enabling social contacts within the neighborhood. For example, Cho et al. [65] concluded that increased accessibility of the home environment was associated with increased health and social outcomes, while a lack of accessibility resulted in negative outcomes, such as becoming housebound.

On the macro level, financial resources, such as receipt of a sufficient pension to finance the goods and services required by the participants, appeared to be a balancing factor for multidimensional frailty. Related to this, Peek et al. [66] demonstrated that financial concerns lead to an increased risk of developing frailty in later life. Nonetheless, our preliminary findings suggest that financial strains may lead to less positive frailty outcomes (e.g., a lower self-reported QoL), rather than an increased risk of frailty. A second finding regarding macro-level factors, is that some older people expressed that they were not able to adapt to digital changes in society, which seemed to influence their frailty balance in a negative way. Related to this might be the concept of “financial fragility,” as introduced by Lusardi and Mitchel [67]. These researchers point to the challenge of lack of financial know-how to manage the complexity of new financial products using digital tools, which eventually leads to a certain level of financial fragility [67]. In addition, health literacy seemed to be an important macro-level factor. Previous research has shown that, especially among older adults, inadequate health literacy is associated with poorer physical and mental health [68–70]. Lastly, care provided by healthcare organizations is an important macro-level factor. Whilst most people received formal care, the social aspect of it (e.g., being able to tell someone about your problems) seemed to have a positive effect on the frailty balance.

This study endorses the argument that two individuals with the same level of frailty can vary greatly in terms of the kind of additional support that they need, because of differences in their frailty balance [24], particularly because their levels of resources to cope with their frailty may differ. Hence, individuals who do not have sufficient balancing factors (resulting in a negative frailty balance) may be more in need of care and assistance than others.

The third research aim concerned the exploration of turning points and their effect on (perceived) frailty and positive outcome variables. The study revealed that general types of adverse life events, such as the death of a loved one, are experienced differently by individual older adults. Some older people also identified positive life events (rather than only adverse life events), such as the birth of grandchildren, as playing a role in their (perceived) frailty and positive outcome variables. Other life events, such as a divorce, may also be experienced in various ways by different individuals: while one individual experienced it as an adverse life event, another perceived it as a positive experience. In addition, previous research has shown that adverse life events are often associated with sudden events such as (personal) illness or death of a loved one [71]. The results of this study add to the literature by demonstrating that life events (e.g., illness of a spouse) can appear gradually as well as suddenly.

### Strengths and limitations

In this study, some limitations that warrant further consideration and research should be noted. First, nine researchers conducted the interviews, which can be seen both as a limitation (e.g., there might have been inconsistencies between interviews) and a strength (e.g., different viewpoints were taken into account, increasing the validity of the study) [72]. However, potential differences were mitigated as far as possible by training and regular meetings between the researchers. Experiences were shared and difficulties discussed, following the QUAGOL methodology [45]. Second, by assessing ‘overall’ QoL, care and support, meaning in life, and mastery, it could be that participants had different definitions in mind [73]. However, each construct was explained, and 1-item measures have been used to measure these constructs in previous studies (i.e. [44, 45, 46]). During this study, we opted using minimal questions QoL, care and support, meaning in life, and mastery due to the vulnerable population we targeted to interview, however, we are well aware that using scales with more items is better but appeared to difficult with frail older adults.

Third, although people with the diagnosis of moderate or severe dementia were excluded in our study, it cannot be stated with certainty that none of the participants had cognitive impairment. Some of the participants scored rather low on the MOCA and therefore might have Mild Cognitive Impairment or (mild) dementia, which could bias the aforementioned results. However, previous research has indicated that many people in population-based cohorts score rather low on the MoCA [74].

Fourth, the attendance of spouses or other relatives during the interviews, although limited, could have generated bias, since their presence could have influenced respondents towards giving socially desirable responses [75]; however, although the researchers emphasized that the interview was best held in private, as the interviews were held in each participant’s private home, and the researchers could not force household members out of the room. Related to this, an interpreter was present during the interviews involving people with a migration background who were not able to express themselves in Dutch or French. This might have led to a bias between the translation of the answers by the interpreter and interpretation by the researchers. Finally, due to the exploratory design of the study, no causal relationships could be established.

This study also has a number of strengths. First, we used a mixed-methods, explorative study design, which contributed to the quality of our data [76]. Second, we focused on the strengths and resources of the included individuals and not solely on negative outcomes. Third, including people with a migration background (from multiple countries) contributes to the generalizability of the study to different regional settings. Fourth, this study proves that it is possible to interview a large number of (frail) older people. The collaboration with professionals from local organizations (e.g., general practitioners, nurses, and other homecare professionals) was essential as they acted as gateways to reach the required population. The (potentially frail) participants were selected purposively to ensure that the diversity of the target population was reflected and, in particular, to ensure coverage of all the experiences of frail, older adults.

## Conclusions

This study was designed to gain insights into the lived experiences associated with frailty and to focus on the strengths that (frail) older adults have. Our quantitative and qualitative findings show that most frail, older adults report having a good QoL, generally receive sufficient care and support, and had relatively high levels of meaning in life and mastery, although further research is needed to explore in-depth why a small subgroup scored lower on these outcome measures. The relationship between possible balancing factors and the outcome measures could be explored more in-depth by a longitudinal study.

Based on the insights gained into the strengths that older people have, which are important for their QoL (amongst other factors), public policymakers and healthcare organizations should promote care and support for older people using a strengths-based approach, rather than a solely deficit-based model. Our study shows that it is crucial to gain insights into the competences and resources that frail, older adults retain, instead of perceiving frailty in older people as entirely negative and frail, older adults as dependent.

## Abbreviations

BAS: Belgian ageing studies
CFAI: Comprehensive frailty assessment instrument
D-SCOPE: Detection, aupport and care for older people, prevention and empowerment
MAXQDA: Qualitative data analysis software
QoL: Quality of life
QUAGOL: Qualitative analysis guide of Leuven
SPSS: Statistical package for the social cciences
